# Cooperation, communication, and co-evolution: grand challenges in microbial symbiosis research

**DOI:** 10.3389/fmicb.2014.00164

**Published:** 2014-04-10

**Authors:** Nicole S. Webster

**Affiliations:** Australian Institute of Marine ScienceTownsville, QLD, Australia

**Keywords:** symbiosis, microbe, host, challenge, holobiont, meta-organism, co-evolution

Microorganisms form symbiotic partnerships with eukaryotes that span all evolutionary stages, from simple amoebae through to humans. The term symbiosis originates from the Greek word “Symbioun” meaning “to live together” and was defined by Anton deBary in 1879 as “the living together of two dissimilar organisms, usually in intimate association, and usually to the benefit of at least one partner” (De Bary, 1879). Whilst the original deBary definition encompasses pathogens and commensals, the term “microbial symbiont” is most commonly used to describe a microorganism that forms a mutualism—a specific, stable and beneficial association with its host (Nyholm and Graf, 2012). Importantly, symbiotic members are “partners” and whilst the term “host” may imply that one partner accommodates or facilitates the association, both members of microbial mutualisms actively contribute to the relationship and the term “host” merely indicates the larger partner. This Grand Challenge Article briefly summarizes the current state of microbial symbiosis research and identifies the methodological and conceptual challenges facing the field into the future.

Microbial symbioses are generally categorized as parasitism, commensalism, or mutualism, though some relationships may wander across these defined boundaries depending on evolutionary processes, changes in environmental conditions and/or health state of the host/symbiont. These include cases such as chlamydia where microbes initially infect hosts as pathogens but over time have evolved mechanisms that assist their persistence and ultimately the survival of their host (Horn et al., 2004). Microbial parasitism has been (and is still) a primary focus of research into symbiotic interactions largely due to the adverse impacts pathogens have on human health and the deleterious effects they have on agricultural and animal stocks. However, beneficial microbial infections whereby hosts gain resources or services that enhance their fitness are known to be just as ubiquitous as parasitic ones and are rapidly gaining research traction. Research on both parasitic and mutualistic associations provide insights into the mechanisms of host-symbiont interactions, such as recognition / specificity adaptations etc., and challenges for the field of microbial symbiosis research can therefore largely be considered independent of the nature of the interaction.

Symbiosis research has historically focused on associations that (i) have economic importance, e.g., nitrogen fixing rhizobia in commercial legume species (Gage, 2004), (ii) have implications for human health, e.g., *Helicobacter pylori* which is responsible for duodenal and gastric ulcers in humans or (iii) offer ecologically fascinating insights, e.g., *Wolbachia* that can significantly alter the reproductive capabilities of insect hosts (Serbus et al., 2008), bioluminescent vibrio that occupy the light organ of bobtail squid and provide luminescence for host feeding and camouflage (McFall-Ngai, 2008b), chemoautotrophic symbionts that convert compounds like hydrogen sulfide and carbon dioxide into organic molecules on which their deep sea hydrothermal vent hosts can feed (Dubilier et al., 2008) and zooxanthellae which contribute photosynthates critical for growth and survival of their coral hosts (Muscatine and Porter, 1977). Many of these examples involve only one, or relatively few, microbial symbionts and are therefore tractable models for obtaining insights into host symbiont ecology and evolution. Investigations of these low microbial diversity systems have greatly enhanced our understanding of mechanistic interactions, such as molecular communication and co-metabolism, between the symbiotic partners (Dale and Moran, 2006). Novel evolutionary principles such as genome reduction, which facilitates a transition of the symbiotic relationship from facultative to obligate, have also been revealed by comparative genome analysis of single symbiont systems (Moran, 2002; Moran et al., 2008). However, microbial symbioses range in complexity from those with a single microorganism to those with many hundreds or thousands of obligate or facultative symbionts, e.g., termite hindgut (Hongoh, 2011), human gut (Marchesi, 2010) and marine sponges (Webster and Taylor, 2012). The development of next generation sequencing (NGS) methods has intensified research into these more complex microbial symbiont communities, facilitating analysis of both diversity and functionality.

The vast majority of microbial symbionts are not amenable to traditional cultivation methods and the application of genetic and genomic approaches has therefore dramatically accelerated research in the field of microbial symbiosis (McFall-Ngai, 2008a). NGS projects are producing an almost overwhelming amount of data and this revolution in delivery of molecular information has fundamentally altered our understanding of microbial symbiosis. As an increasing number of host-associated microbial environments are explored using NGS, estimates of microbial diversity have exploded [e.g., a recent analysis of ascidian species documented 3217 unique bacterial OTU'S, (Erwin et al., 2014)], unexpected genomic features have been uncovered [e.g., extreme genome reduction has been reported in a wide range of bacterial symbionts (McCutcheon and Moran, 2012)], functional equivalence and evolutionary convergence has been reported in complex symbiont communities (Fan et al., 2012) and unexpected patterns of host-specificity have been revealed [e.g., previously considered sponge—specific bacterial sequences have now been recovered from the rare biosphere of diverse marine environments (Taylor et al., 2013)]. These studies herald an era of unprecedented discovery in symbiosis research yet also reveal the daunting scale of the task ahead in deciphering the forces that drive relationships within these complex symbiotic systems.

Lifestyles of bacterial symbionts can vary in four important ways, all of which contribute to the long term evolution of symbiotic microbial lineages as well as the co-evolution of the holobiont: (1) host-symbiont specificity, (2) the mechanisms of symbiont acquisition, development and maintenance, (3) the functional mechanisms that the symbiont employs to infer benefit or detriment to the host, e.g., nutrient transport, chemical defense etc. and (4) the host response to infection by the symbionts. Specificity varies greatly ranging from strictly obligate associations, e.g., aphids harbor *Buchnera aphidicola* which provide essential amino acids that are lacking in their diet (Shigenobu et al., 2000) through to highly facultative symbioses, e.g., the diverse and temporally dynamic symbionts that occur in some marine sponges, (Webster et al., 2011). Importantly, selection for the establishment and persistence of the symbiosis can occur by either or both of the symbiotic partners and adaptations for recognition and colonization are often found in both the host and symbiont. The symbiosis between the squid *Euprymna scolopes* and its bioluminescent bacterial symbiont *Vibrio fischeri* is a classic example of “winnowing”- a gradual elimination of potential light organ colonizers that ensures separation of the specific strain of symbiotic *V. fischeri* from the milieu of environmental microbes present in the seawater (Nyholm and McFall-Ngai, 2004). The evolution of symbiont-specific factors for colonization was also recently highlighted by analysis of *Bacillus* in the mouse gut where novel molecular mechanisms in the symbiont were found to control the specificity and stability of other gut microbiota (Lee et al., 2013).

In terms of host acquisition, symbionts can be acquired (i) horizontally from the environment, e.g., from food in the human gut (Ley et al., 2008) or contemporaries, e.g., “egg smearing,” in stinkbugs which involves the female contaminating the surface of her eggs with symbiont-laden feces during oviposition (reviewed in Funkhouser and Bordenstein, 2013), (ii) vertically from parental inheritance or (iii) via a combination of these mechanisms (Bright and Bulgheresi, 2010). Horizontal symbiont transmission often leads to selection based on symbiont function rather than symbiont taxonomy (Turnbaugh and Gordon, 2009; Burke et al., 2011). Establishing horizontally acquired symbioses presents considerable challenges for both the host and the symbiont. Mechanisms are required that enable the selection and retention of specific microbes from the environment whilst at the same time the host needs to retain normal functioning of the immune system to enable them to destroy opportunistic or potentially pathogenic microorganisms (reviewed in Bright and Bulgheresi, 2010 and references therein). Whilst our understanding of the physiological requirements, nutritional conditions and immune system factors required for the establishment and maintenance of horizontally acquired symbioses in non-model species is rudimentary, in some models such as the bobtail squid-vibrio symbiosis, these parameters have been very well established (reviewed in Nyholm and Graf, 2012). Exploration and detailed understanding of these model systems will undoubtedly help direct researchers in deciphering the nature of symbiotic interactions in other non-model species.

Symbionts can also be transmitted vertically through reproductive cells and larvae, as has been demonstrated in insects (Moran and Baumann, 2000), ascidians (Kojima and Hirose, 2012), sponges (Usher et al., 2001; Webster et al., 2010) and a diverse range of other higher organisms (McFall-Ngai, 2002). In contrast to the phylogenetically diverse communities that can establish a symbiosis through horizontal acquisition, vertical transmission generally leads to more streamlined microbial communities with reduced taxonomic and functional complexity. A classic example of vertical transmission is the symbiosis between the pea aphid *Acrythosiphon pisum* and its nutritional endosymbiont *Buchnera aphidicola*. This symbiosis was established over 160 million years ago and is maintained through strict vertical transmission (Baumann, 2005). The endosymbionts are contained within bacteriocytes in a region of the aphid body cavity that allows their successful transfer to developing oocytes or embryos during aphid reproduction (Baumann et al., 1995). This mutualism is so obligate that neither the host nor the symbiont can reproduce independently. Genome analysis of the symbiont revealed genes for biosyntheses of amino acids required by the host and an absence of non-essential amino acids, indicating complementarity and syntrophy between the host and the symbiont (Shigenobu et al., 2000). Co-evolution of the host and symbiont in many of these vertically transmitted symbioses often leads to obligate relationships where the symbionts begin to resemble organelles (Moran, 2006; Russell et al., 2013).

As our knowledge of symbiosis continues to expand, so too does our understanding of how symbiosis has contributed to evolution. It is thought that over 1 billion years ago the evolution of eukaryotic organelles—the plastids and mitochondria—occurred from *Cyanobacteria* and *Alphaproteobacteria* respectively (Dyall et al., 2004). In establishing this symbiosis the endosymbionts would have lost core parts of their genomes, resulting in the loss of much of their bacterial identity, and acquired many host-derived properties as they transformed into organelles comprising essential components of eukaryotic host cells (Dyall et al., 2004). Symbiotic interactions with microorganisms continued to contribute to evolution of the ancestors of extant organisms by opening up new avenues for nutrition, defense and niche occupation, e.g., it is believed that arbuscular mycorrhizal fungi played a major role in the colonization of land by plants (Redecker et al., 2000). Interleaving of host and symbiont genomes can increase the metabolic potential of the host, thereby increasing its niche range, ecological flexibility and adaptation potential. Clear examples of this are found across many invertebrate species hosting intracellular symbionts whose genes encode for functions like the synthesis of essential amino acids (Baumann, 2005), photosynthesis (Venn et al., 2008) or chemosynthesis (Dubilier et al., 2008). These microbial-derived metabolic functions enable the host to successfully inhabit novel environments or adopt alternative lifestyles that would otherwise not be possible. Co-evolution of animals and microbes has also guided the distribution and diversification of bacteria, with some species not detected outside their host environment (Hongoh, 2010).

The terms “holobiont” or “metaorganism” (Bosch and McFall-Ngai, 2011) are increasingly being cited by researchers from a range of scientific disciplines to describe the complex communities comprised of the animal / plant host and the diverse array of bacteria, archaea, fungi, algae and viruses that associate with it [the microbiome, (Hooper and Gordon, 2001)]. However, there is still a tendency by scientists to view the symbiotic partners as separate individuals which can limit our ability to assess interactive mechanisms, including synergism, within these holobiont systems. As highlighted by recent studies of the human microbiome, properties of a metaorganism are often not adequately explained by the isolated features of its individual elements (Relman, 2008). Overcoming this perception of individualism is required if we are to truly understand the ecology and evolution of microbial symbioses.

In a recent focus article, the eminent human microbiome researcher David Relman surmises that “Symbioses are the ultimate examples of success through collaboration and the powerful benefits of intimate relationships” (Relman, 2008). This eloquent expression emphasizes the advantages that cooperation with microbial partners bring to all life forms. The challenge in moving beyond the exploratory phases of symbiosis research, e.g., documenting the diversity of microbes present in different host species, will be to unite interdisciplinary researchers to pose novel questions about the nature of the species interactions. For example, what role do physical forces (e.g., ocean currents), chemical cues (e.g., chemotaxis) and interspecies communication (e.g., quorum sensing) play in the establishment and maintenance of symbiosis for various marine species? With major advances in both molecular and imaging technologies, we are well poised to better understand these types of questions. Additional aspects of symbioses that still need to be elucidated include: (i) the many mechanisms that underpin how microbial symbioses are established, evolve and are maintained over time, (ii) how microbial symbiosis influences host development, fitness and survival and (iii) whether microbial symbionts can assist their partners in adapting to rapidly changing environments and vice versa. Each example of microbial symbiosis offers unique insights into the above questions yet is constrained by its own set of limitations. Whilst sequence based approaches are invaluable for generating new hypotheses, these in turn still need to be validated by detailed functional experiments. Only by applying a diverse range of methodological approaches to a broad suite of model and non-model systems studied by a well-networked community of interdisciplinary researchers will we truly be able to reveal the extraordinary extent of symbiotic interactions that exist in nature.

## Conflict of interest statement

The author declares that the research was conducted in the absence of any commercial or financial relationships that could be construed as a potential conflict of interest.
